# Aging Leads to Increased Monocytes and Macrophages With Altered CSF-1 Receptor Expression and Earlier Tumor-Associated Macrophage Expansion in Murine Mesothelioma

**DOI:** 10.3389/fragi.2022.848925

**Published:** 2022-04-27

**Authors:** Lelinh Duong, Fiona J. Pixley, Delia J. Nelson, Connie Jackaman

**Affiliations:** ^1^ Curtin Medical School, Faculty of Health Sciences, Curtin Health Innovation Research Institute, Curtin University, Perth, WA, Australia; ^2^ School of Biomedical Sciences, The University of Western Australia, Perth, WA, Australia

**Keywords:** macrophage, monocyte, CSF-1R, aging, cancer, tumor-associated macrophages

## Abstract

Increased cancer incidence occurs with the emergence of immunosenescence, highlighting the indispensability of the immune system in preventing cancer and its dysregulation with aging. Tumor-associated macrophages (TAMs) are often present in high numbers and are associated with poor clinical outcomes in solid cancers, including mesothelioma. Monocytes and macrophages from the bone marrow and spleen can respond to tumor-derived factors, such as CSF-1, and initiation of the CSF-1R signaling cascade results in their proliferation, differentiation, and migration to the tumor. Age-related changes occur in monocytes and macrophages in terms of numbers and function, which in turn can impact tumor initiation and progression. Whether this is due to changes in CSF-1R expression with aging is currently unknown and was investigated in this study. We examined monocytes and macrophages in the bone marrow and spleen during healthy aging in young (3–4 months) and elderly (20–24 months) female C57BL/6J mice. Additionally, changes to these tissues and in TAMs were examined during AE17 mesothelioma tumor growth. Healthy aging resulted in an expansion of Ly6C^high^ monocytes and macrophages in the bone marrow and spleen. CSF-1R expression levels were reduced in elderly splenic macrophages only, suggesting differences in CSF-1R signaling between both cell type and tissue site. In tumor-bearing mice, Ly6C^high^ monocytes increased with tumor growth in the spleen in the elderly and increased intracellular CSF-1R expression occurred in bone marrow Ly6C^high^ monocytes in elderly mice bearing large tumors. Age-related changes to bone marrow and splenic Ly6C^high^ monocytes were reflected in the tumor, where we observed increased Ly6C^high^ TAMs earlier and expansion of Ly6C^low^ TAMs later during AE17 tumor growth in the elderly compared to young mice. F4/80^high^ TAMs increased with tumor growth in both young and elderly mice and were the largest subset of TAMs in the tumor. Together, this suggests there may be a faster transition of Ly6C^high^ towards F4/80^high^ TAMs with aging. Amongst TAM subsets, expression of CSF-1R was lowest in F4/80^high^ TAMs, however Ly6C^low^ TAMs had higher intracellular CSF-1R expression. This suggests downstream CSF-1R signaling may vary between macrophage subsets, which can have implications towards CSF-1R blockade therapies targeting macrophages in cancer.

## Introduction

Advancing age is associated with changes to the immune system, described as immunosenescence (Pawelec and Solana, 1997; Pawelec, 2012). A chronic, low-grade increase in circulating inflammatory factors is also associated with aging, known as inflammaging (Franceschi et al., 2000; Franceschi et al., 2007) and is a likely contributing factor to immunosenescence (Pawelec, 2012; G.; Pawelec and Solana, 1997). Moreover, increased cancer incidence occurs with the emergence of immunosenescence, highlighting the indispensability of the immune system in preventing cancer and its degradation with aging.

Tumor-associated macrophages (TAMs) are often present in high numbers and are associated with poor clinical outcomes in solid cancers (Zhang et al., 2013; Yang et al., 2018; Ding et al., 2019; Yagi et al., 2019). Monocytes are recruited to the tumor from the bone marrow in response to chemotactic signals (Sanford et al., 2013; Shand et al., 2014). In chronic inflammation, monocytes can also be recruited to the tumor from the spleen (Cortez-Retamozo et al., 2012; Cortez-Retamozo et al., 2013; Shand et al., 2014). Tumour infiltrating monocytes are predominantly Ly6C^high^ and can be recruited *via* CSF-1/CSF-1R signaling (Ryder et al., 2013; Ries et al., 2014; Wang et al., 2016; Gyori et al., 2018). As well as stimulating chemotaxis, CSF-1 is a primary growth factor for macrophages and subsequently drives differentiation of myeloid progenitor cells into mature macrophages (Pixley and Stanley, 2004). Following CSF activation, the CSF-1R is internalized and eventually degraded (Guilbert et al., 1986; Kanagasundaram et al., 1998; Lou et al., 2014). Monocytes maturing into TAMs can be distinguished through differential expression of Ly6C and F4/80, with downregulation of Ly6C and upregulation of F4/80 (Shi and Pamer, 2011; Ramachandran et al., 2012; Crane et al., 2014; Franklin et al., 2014).

Age-related changes to monocytes/macrophages could impact tumor initiation and progression. For example, studies have shown a shift towards myelopoiesis with healthy aging (Rossi et al., 2005; Beerman et al., 2010; Pioli et al., 2019), which may lead to a larger pool of monocytes available for recruitment during tumor development. This is supported by our previous study, which showed higher numbers of macrophages in mesotheliomas from elderly compared to young mice (Duong et al., 2018). Function may also be impacted by aging as murine bone marrow-derived macrophages and human peripheral blood mononuclear cell-derived macrophages from aged cohorts stimulated *ex vivo* with LPS increased levels of pro-inflammatory cytokines such as TNF and IL-6 (Bouchlaka et al., 2013; Barrett et al., 2015; Thevaranjan et al., 2017). In contrast, splenic macrophages from elderly mice stimulated with LPS had reduced TNF, IL-6 and IL-1β production compared to young mice (Gomez et al., 2010; Mahbub et al., 2012). Splenic and peritoneal macrophages from elderly mice also exhibit reduced phagocytosis (Linehan et al., 2014; Tomay et al., 2018). Whether this is due to changes in CSF-1R signaling with aging is currently unknown. Interestingly, two cytokines associated with inflammaging, TNF and IFN-γ, have been shown to downregulate CSF-1R expression (Rovida et al., 2001; Delneste et al., 2003) and circulating CSF-1 is also increased during aging (Suehiro et al., 1999; Larsson et al., 2015; Lira-Junior et al., 2017). Therefore, inflammaging could impact monocyte/macrophage differentiation and CSF-1R expression during aging and cancer.

Given that monocyte/macrophage numbers increase in elderly healthy mice, as do TAMs in tumors from elderly mice (Jackaman et al., 2013), their infiltration into tumors may be mediated by increased CSF-1/CSF-1R signaling. Moreover, altered macrophage responses to pro- and anti-inflammatory stimuli combined with the inflammaging microenvironment may further alter their response. Therefore, this study first investigated changes to monocyte/macrophage proportions and CSF-1/CSF-1R signaling in bone marrow and spleen during healthy aging. We then assessed whether the balance was altered during tumor growth in a murine mesothelioma model, a CSF-1 secreting cancer predominantly found in the elderly (Bianchi and Bianchi, 2007; Magkouta et al., 2021).

## Materials and Methods

### Animal Model

Female C57BL/6J mice were obtained from the Animal Research Centre (ARC, Murdoch, WA, Australia) and maintained at Curtin University animal facilities under specific-pathogen free conditions. Young mice were aged 3–4 months (equivalent to 18 year-old humans) and elderly mice were 20–24 months (60–70 year-old humans), as defined by the Jackson Laboratory (Yuan et al., 2009). Mice were excluded from the study if they had enlarged organs, a palpable mass or experienced excessive body weight loss (>15% from age 12 months) prior to tumor inoculation. Animals were housed in a standard light/dark cycle and fed a standard chow diet ab libitum. Experiments were performed as per the Curtin University Animal Ethics Committee (AEC) in accordance to the Australian Code of Practice for the use and care of animals for scientific purposes (AEC approval numbers: AEC_2016_04 and AEC_2020_03).

### AE17 Murine Mesothelioma Cell Lines

AE17 is a murine malignant mesothelioma cell line generated by inoculation of asbestos fibers and was derived from orthotopic tumor deposits that emerged in elderly C57BL/6J mice and is histologically representative of human mesothelioma (Jackaman et al., 2003). Mesothelioma cell lines, including AE17, have also been reported to express CSF-1 (Fox et al., 2012; Cioce et al., 2014). Cells were maintained in complete medium, containing RPMI 1640 (Invitrogen) supplemented with 10% HyClone™ fetal bovine serum (FBS, Cytiva, Utah, America), 2 mM L-glutamax (Life Technologies, Victoria, Australia), 100 units/ml of penicillin, 100 μg/ml streptomycin (Life Technologies) and 0.05 mM 2-mercaptoethanol (Sigma-Aldrich) at 37°C with 5% CO_2_. Cells were collected for tumor inoculation when ≥80% confluent. Mice were inoculated subcutaneously (s.c.) with 5 × 10^5^ cells in 100 μl PBS and body weight, body condition score and tumors monitored daily. Tumor sizes were taken daily using calipers and determined by measurement of tumor width (mm) and length (mm), and calculated as width × length in mm^2^. Each mouse was tracked individually for tumor growth. Mice were monitored until their individual maximum tumor size reached 140 mm^2^, weight loss exceeded >20% or reached experimental endpoint. Based on our previous studies, age impacted tumor growth in that tumors grew faster in elderly compared to young mice (Duong et al., 2018). In order to control for variation between young and elderly tumor growth, samples were collected based on tumor size. Tumors were collected at either early (7–10 days post s.c. injection) or late timepoints (18–23 days post s.c. injection); small tumors measured <30 mm^2^ and large tumors were between 65 and 140 mm^2^ (Jackaman et al., 2003; Jackaman et al., 2011; Jackaman et al., 2016).

### Flow Cytometry

To ensure temperature did not impact CSF-1R expression, all reagents were pre-cooled on ice and samples kept on ice throughout staining. Sample collection was also timed so that young and elderly samples spent the same amount of time in processing *ex vivo* once tissues were out of the animal. Similar numbers of young and elderly samples were also included in each flow cytometry experiment to control for any inter-assay staining variation. Bone marrow, spleen and tumors were collected into collection/staining buffers: ice-cold phosphate buffered solution (PBS) containing 2% fetal bovine serum and 2 mM EDTA (Sigma-Aldrich). Tibiae and femurs were flushed with a 29 g needle to isolate and create single cell suspension of bone marrow cells. Spleens and tumors were disaggregated gently into single cell suspension between two frosted slides in staining buffer. For flow cytometry surface staining, single cell suspensions were blocked with anti-mouse CD16/32 (clone 93, Biolegend) for 15 min on ice, all subsequent steps were performed on ice in the dark unless otherwise stated. A combination of anti-mouse antibodies diluted in staining buffer were incubated for 30 min followed by PBS and incubated for 15 min with Zombie-NIR™ (Biolegend). Intracellular staining was performed after fixing and permeabilization with True-Nuclear™ Transcription Factor Buffer Set (Biolegend) as per manufacturer’s instructions. Surface and intracellular antibodies were diluted in 1x Transcription Factor permeabilization buffer for 1 h in the dark at room temperature. A combination of the following anti-mouse antibodies were used: anti-CD11b BUV395 (BD) or anti-CD11b Alexafluor^®^ 488 (clone M1/70, Biolegend), Ly6C BV510 (clone HK1.4, Biolegend), anti-Ly6G Brilliant Violet 785™ (clone 1A8, Biolegend), anti-F4/80 PE-Dazzle-594 (clone BM8, Biolegend) or anti-F4/80 Alexafluor^®^ 647 (clone BM8, Biolegend), anti-CD115 (CSF-1R) Brilliant Violet 421™ (clone AFS98, Biolegend) and anti-CD206-PE-Cy7 (clone C068C2, Biolegend). After staining, cells were washed twice and resuspending in staining buffer for acquisition on BD LSRFortessa™ flow cytometer using FACSDiva (BD Biosciences). Unstained, single stains and fluorescent minus one controls were used for instrument setup, compensation and analysis. Data was analyzed using FlowJo version 10.7 (BD Bioscience).

### Measurement of Circulating CSF-1 Levels

Whole blood was collected directly into EDTA (Sigma-Aldrich) and plasma stored at −20°C until analysis. Samples were collected from young healthy mice, elderly healthy mice, large tumor-bearing young mice and large tumor-bearing elderly mice. The concentration of CSF-1 in plasma samples was measured using a LEGENDplex™ Mouse Hematopoietic Stem Cell Panel (Biolegend) as per manufacturer’s instructions. Data was collected on a LSRFortessa™ flow cytometer using FACSDiva (BD Biosciences) and analyzed using FlowJo version 10.7 (BD Bioscience).

### Statistical Analysis

GraphPad Prism version 9 (California, CA, United States) was used to analyze data. Data presented as means ± SEM. Mann-Whitney *U*-test was used to determine differences between two populations. A relationship between two variables was determined by Pearson’s correlation coefficient test. *p-*values of <0.05 were considered statistically significant.

## Results

### Healthy Aging Leads to Expansion of Monocytes and Macrophages in the Bone Marrow and Spleen

**FIGURE 1:**
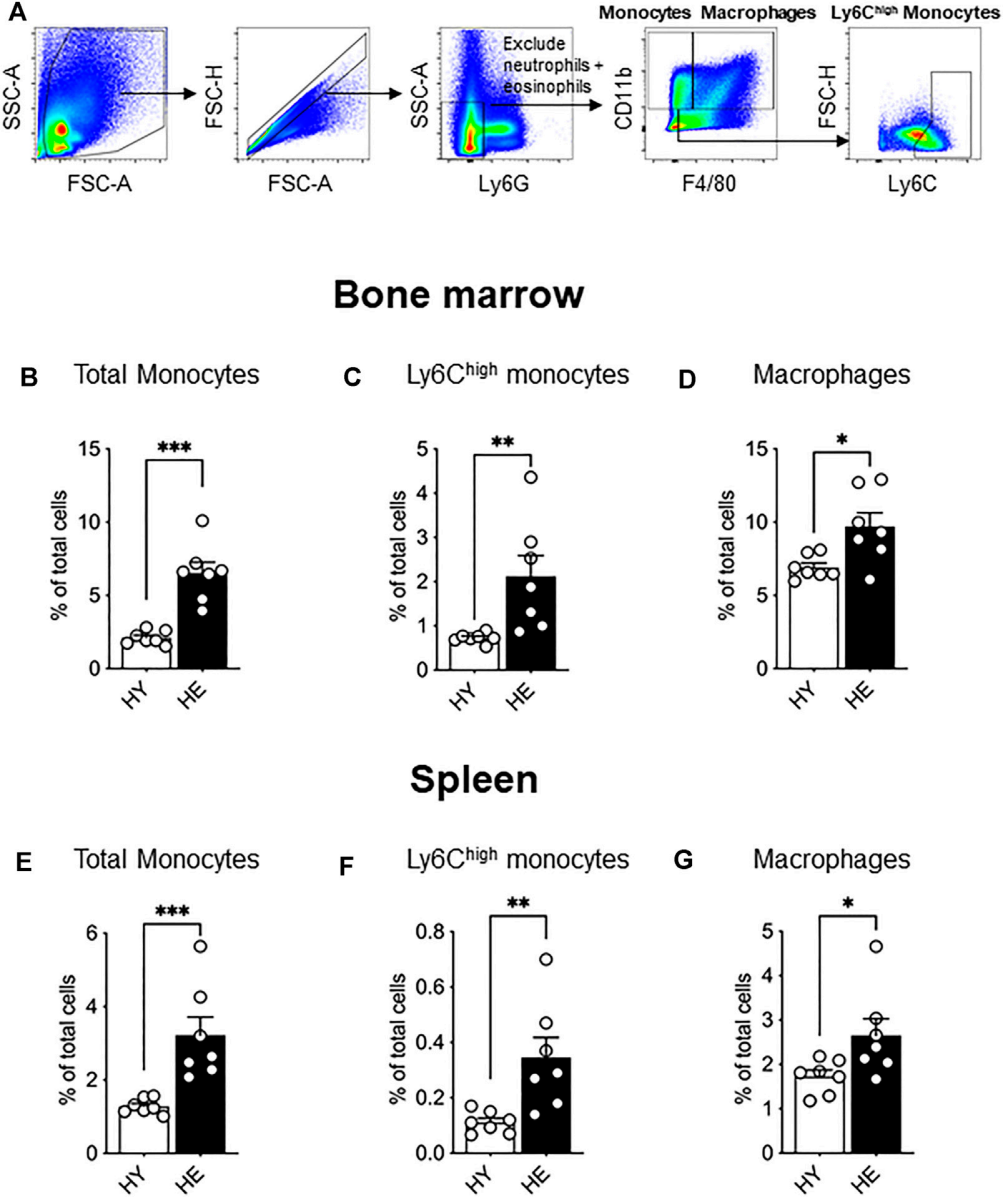
Bone marrow and splenic monocytes and macrophages increased with healthy aging. Bone marrow and spleen from healthy, non-cancer-bearing mice were dissociated into single cell suspension and stained for CD11b, F4/80, Ly6G and Ly6C and analyzed *via* flow cytometry. Macrophages were recognized as CD11b^+^F4/80^+^Ly6G^neg^ and total monocytes as CD11b^+^F4/80^neg^Ly6G^neg^ with Ly6C^high^ monocytes further identified [a representative gating strategy shown in **(A)**. Total monocytes, Ly6C^high^ monocytes and macrophages shown as percentage of total cells in the bone marrow **(B−D)**, respectively] and in the spleen [**(E−G)**, respectively]. HY, healthy young; HE, healthy elderly. Data shown as mean ± SEM; *n* = 7 mice/group; *p < 0.05, **p < 0.01, ***p < 0.005.

*Ex vivo* flow cytometry analysis of bone marrow from young and elderly healthy mice (gating strategy shown in Figure 1A) revealed an age-specific expansion of CD11b^+^Ly6G^neg^F4/80^neg^ monocytes (Figure 1B), a Ly6C^high^ subpopulation (Figure 1C) and CD11b^+^F4/80^+^Ly6G^neg^ macrophages (Figure 1D). We next investigated the spleen as it can serve as a site for extramedullary hematopoiesis and contains a reservoir of monocytes that may be deployed to the tumor (Cortez-Retamozo et al., 2012; Cortez-Retamozo et al., 2013). Similar to bone marrow, healthy aging leads to the expansion of total monocytes (Figure 1E), the Ly6C^high^ subpopulation (Figure 1F) and CD11b^+^F4/80^+^Ly6G^neg^ red pulp macrophages (Figure 1G) in the spleen.

### Healthy Aging Leads to Decreased Expression of CSF-1R in Splenic but Not Bone Marrow Macrophages

**FIGURE 2:**
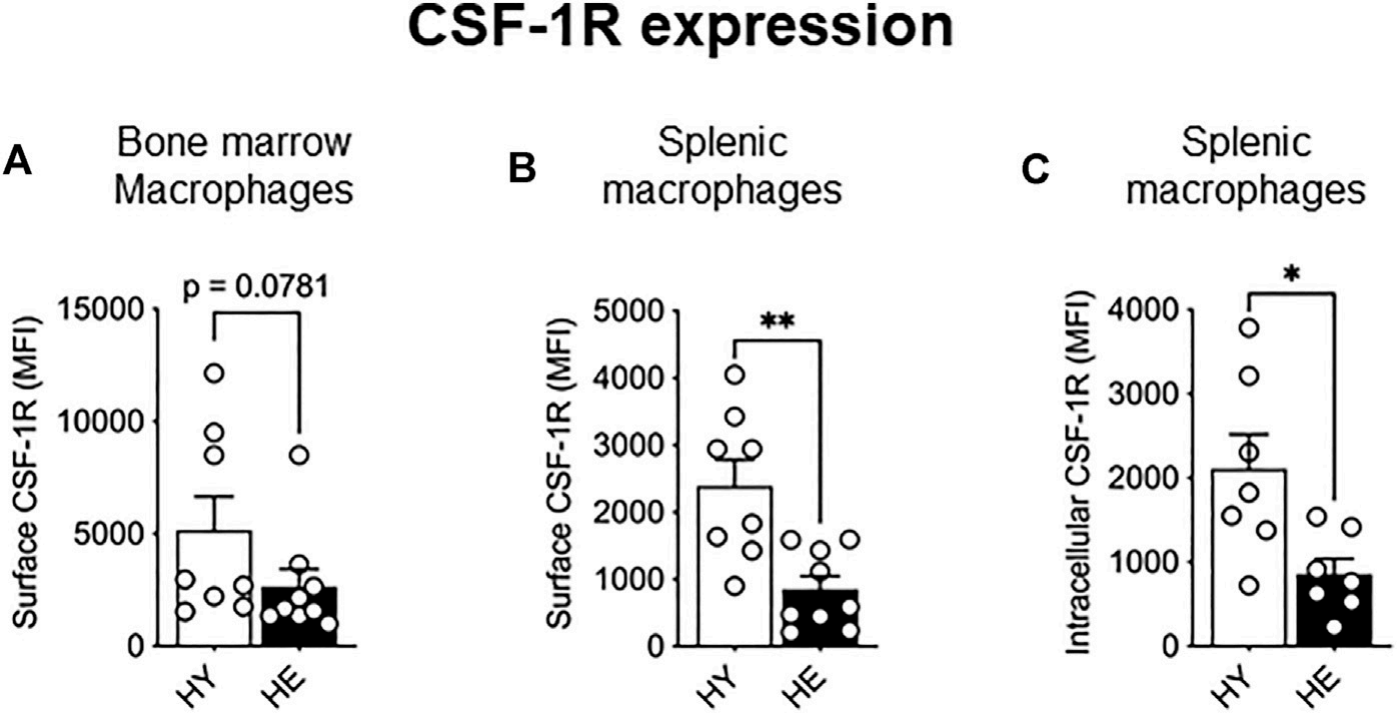
CSF-1R expression decreased in splenic macrophages with healthy aging. CD11b^+^F4/80^+^Ly6G^neg^ macrophage CSF-1R surface expression in the bone marrow **(A)** and spleen **(B)** and intracellular CSF-1R expression in the spleen **(C)** shown as median fluorescence intensity (MFI). HY—healthy young, HE, healthy elderly. Data shown as mean ± SEM; *n* = 7–8 mice/group; *p < 0.05, **p < 0.01.

As CSF-1R signaling is crucial in monocyte to macrophage differentiation, expansion, and migration (reviewed in Pixley and Stanley, 2004), we next measured expression of this molecule to determine whether altered expression levels may account for the increased proportion of these cells in older tissues. While CSF-1R surface and intracellular expression was unchanged between young and elderly healthy macrophage and monocyte populations in the bone marrow (Figure 2A and Supplementary Figure S1) and splenic monocytes (Supplementary Figure S2), a significant age-specific decrease in surface and intracellular CSF-1R was observed in splenic macrophages (Figures 2B,C, respectively). These data show that CSF-1R expression is reduced with healthy aging in splenic macrophages, highlighting age- and tissue-related differential regulation of CSF-1R expression.

### Aging and Cancer Increase Numbers of Both Bone Marrow and Splenic Monocytes

**FIGURE 3:**
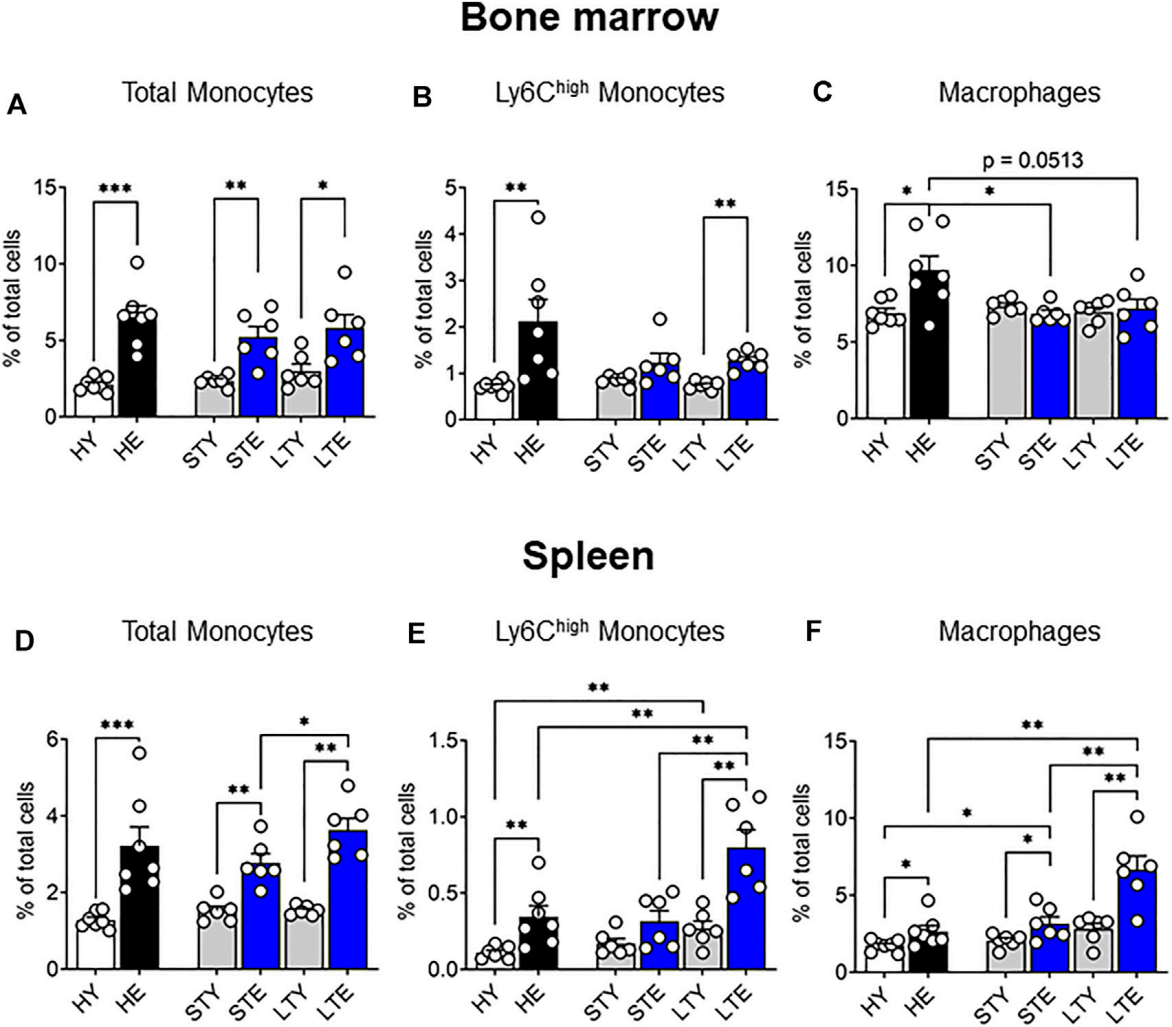
Splenic monocyte and macrophage pools increase with age and is further exacerbated with tumor growth. Young and elderly C57BL/6J mice were inoculated with AE17 mesothelioma tumor cells and sacrificed when tumors were small (early time point, tumor size <30 mm^2^) or large (late time point, tumor size 65–140 mm^2^). Total monocytes (CD11b^+^F4/80^neg^Ly6G^neg^), Ly6C^high^ monocytes and macrophages (CD11b^+^F4/80^+^Ly6G^neg^) shown as percentage of total cells in the bone marrow [**(A−C)**, respectively) and the spleen [**(D–F)**, respectively). Data on healthy mice (Figure 1) have been included here as controls. HY, healthy young; HE, healthy elderly; STY, small tumor young; STE, small tumor elderly; LTY, large tumor young; LTE, large tumor elderly. Data shown as mean ± SEM; *n* = 6–7 mice/group; *p < 0.05, **p < 0.01.

Previously we showed that solid tumors impact the bone marrow macrophage compartment, leading to increased bone marrow macrophage proliferation in elderly-tumor bearing mice compared to young mice (Duong et al., 2018). In this study, we expanded this work to include monocytes with a particular focus on the Ly6C^high^ subset, which infiltrates tumors and differentiates into TAMs (Movahedi et al., 2010; Van Overmeire et al., 2015). As various stages of tumor progression may impact these cells differently, we investigated the bone marrow at small (early stage) and large (late stage) AE17 mesothelioma tumors. Total bone marrow monocytes increased due to aging rather than tumor growth (Figure 3A). Similarly, the proportion of Ly6C^high^ monocytes increased with healthy aging in the bone marrow and was not further affected by tumor growth (Figure 3B). Macrophage proportions remained similar between young and elderly mice bearing tumors (Figure 3C). However, when compared to healthy elderly controls, macrophage proportions were reduced in the bone marrow of elderly mice with cancer (Figure 3C), despite displaying increased proliferation (Duong et al., 2018). It is possible this is due to reduced survival or migration of the cells from the bone marrow to support tumor growth. Next, the splenic monocyte and macrophage compartments were examined during AE17 tumor development. The profile of total splenic monocytes was similar to that seen in the bone marrow with an age-related increase in numbers in small and large AE17 tumors (Figure 3D). However, splenic Ly6C^high^ monocytes increased markedly in elderly mice during tumor progression while their proportions remained steady in young spleens (Figure 3E). This resulted in an almost 3-fold increase in Ly6C^high^ proportions in the elderly compared to young mice with large tumors. Similarly, splenic macrophage proportions were significantly higher in elderly vs. young AE17-bearing hosts and was further augmented with tumour growth (Figure 3F).

### Aging and Cancer Differentially Affect CSF-1R Surface Expression in Monocytes and Macrophages in the Bone Marrow and Spleen

**FIGURE 4:**
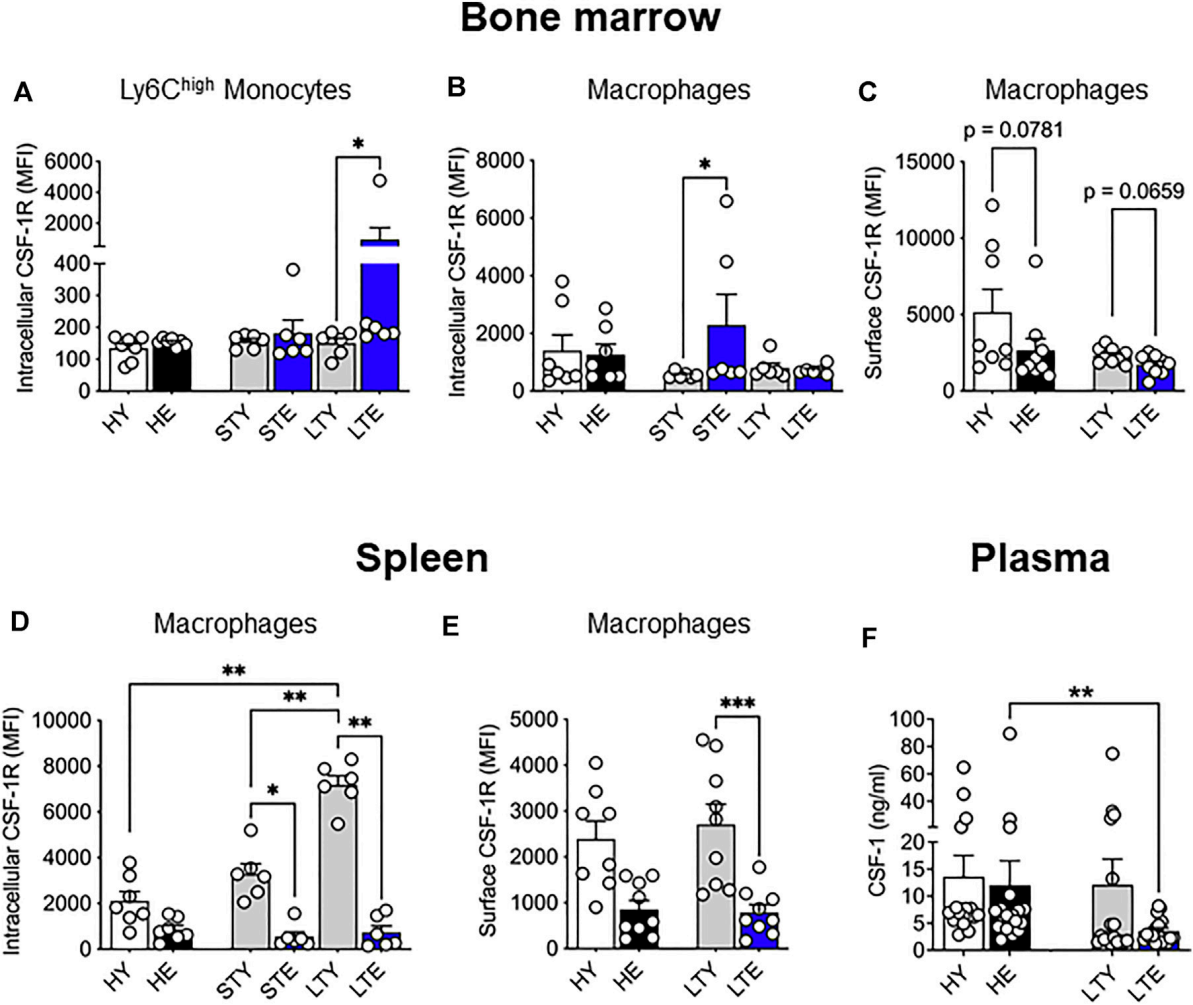
Bone marrow and splenic monocyte and macrophage CSF-1R expression is differentially impacted by aging and cancer. Young and elderly C57BL/6J mice were inoculated with AE17 mesothelioma cells and sacrificed when tumors were at small (early time point, tumor size <30 mm^2^) or large (late time point, tumor size 65–140 mm^2^). Bone marrow Ly6C^high^ monocyte (CD11b^+^F4/80^neg^Ly6G^neg^Ly6C^high^) **(A)** and macrophage (CD11b^+^F4/80^+^Ly6G^neg^) intracellular **(B)** and surface **(C)** CSF-1R expression shown as median fluorescence intensity (MFI). Splenic macrophage intracellular **(D)** and cell surface **(E)** CSF-1R expression shown as MFI. Data on healthy mice (Figure 1) have been included here as controls. Expression shown as median fluorescence intensity (MFI). Circulating CSF-1 levels in plasma samples shown in **(F)**. HY, healthy young; HE, healthy elderly; STY, small tumor young; STE, small tumor elderly; LTY, large tumor young; LTE, large tumor elderly. Data shown as mean ± SEM; *n* = 6–8 mice/group for **(A–E)** and 17–19 mice/group for **(F)**; *p < 0.05, **p < 0.01, ***p < 0.005.

We also examined levels of CSF-1R expression in monocytes and macrophages from the bone marrow and splenic compartments in tumor-bearing mice. Interestingly, intracellular CSF-1R expression in Ly6C^high^ bone marrow monocytes increased in the elderly compared to young with large tumors (Figure 4A), which may reflect increased internalization of activated CSF-1R (Yeung and Stanley, 2003). Furthermore, intracellular CSF-1R expression increased in macrophages of elderly mice during early tumor growth but matched the levels of younger counterparts when tumors were larger (Figure 4B). Surface expression of CSF-1R in bone marrow macrophages was slightly but not significantly reduced in elderly vs. young mice with large tumors (Figure 4C). In the spleen, intracellular CSF-1R expression in total monocytes remained similar through all cohorts and tumor growth (Supplementary Figure S3). However, splenic macrophages in young tumor-bearing mice demonstrated increased CSF-1R expression that was restricted to the intracellular compartment (Figure 4D) and not seen on the cell surface (Figure 4E) when compared to the healthy setting. Intracellular CSF-1R expression in these cells also increased with tumor growth (Figure 4D). These data suggest CSF-1R internalization in young mice during tumor growth. However, there were no changes to CSF-1R expression during tumor growth in elderly mice (and compared to healthy) suggesting splenic macrophages from tumor-bearing mice could be refractory to CSF-1R signaling (Figure 4D,E). These data suggest that CSF-1R expression may be impacted with aging and tumor progression but varies depending on cell type. Previous studies have reported increased circulating CSF-1 during aging (Suehiro et al., 1999; Larsson et al., 2015; Lira-Junior et al., 2017) which could impact the spleen as a blood filter. Furthermore, the AE17 cell line used in this study expresses CSF-1 (Fox et al., 2012). However, we observed no difference in CSF-1 circulating protein levels during healthy aging (Figure 4F) and circulating CSF-1 levels were decreased in the plasma of elderly large tumor-bearing mice compared to elderly healthy controls (Figure 4F). Combined with the overall changes to CSF-1R expression it is possible there is increased utilization of CSF-1 in elderly tumor-bearing mice.

### Aging Leads to Increased Ly6C^high^ Tumor-Associated Macrophages Earlier and Expansion of the Ly6C^low^ Subset Later During AE17 Tumor Development

**FIGURE 5:**
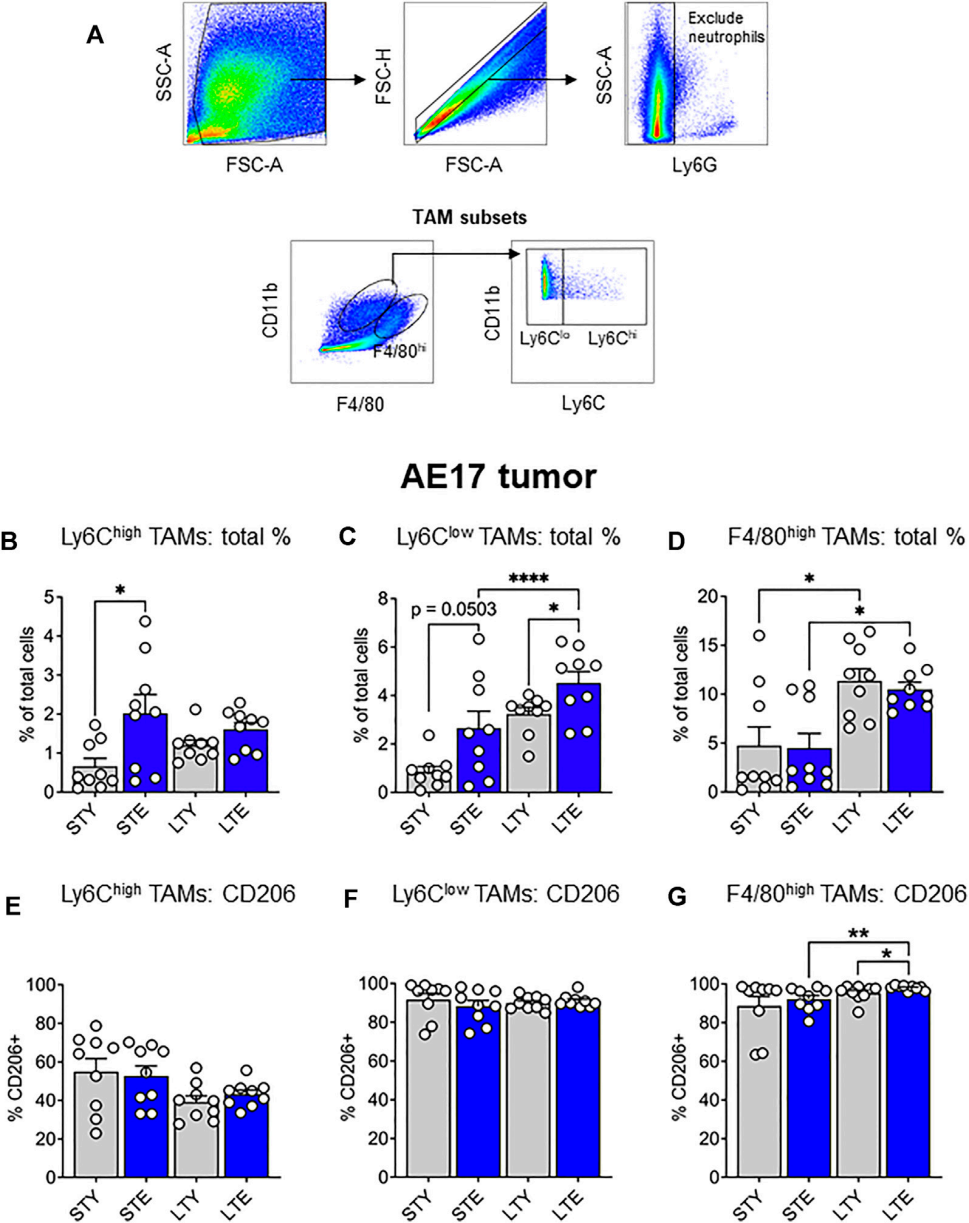
Ly6C^high^ tumor-associated macrophages (TAMs) increased earlier and Ly6C^low^ TAMs later during AE17 tumor development. Young and elderly C57BL/6J mice were inoculated with AE17 mesothelioma cells and sacrificed when tumors were at small (early time point, tumor size <30 mm^2^) or large (late time point, tumor size 65–140 mm^2^). Tumors were dissociated into single cell suspension and stained for CD11b, F4/80, Ly6G and Ly6C and analyzed *via* flow cytometry [a representative gating strategy shown in **(A)**]. The percentage of total cells and intracellular CD206 expression shown for each TAM subset: Ly6C^high^ (CD11b^+^Ly6G^neg^F4/80^int^Ly6C^high^) **(B and E)**, Ly6C^low^ (CD11b^+^Ly6G^neg^F4/80^int^Ly6C^low^) **(C and F)** and F4/80^high^ (CD11b^+^Ly6G^neg^F4/80^high^Ly6C^high/low^) **(D and G)**. STY—small tumor young, STE, small tumor elderly; LTY, large tumor young; LTE, large tumor elderly. Data shown as mean ± SEM; *n* = 9 mice/group; *p < 0.05, ****p < 0.001.

The data presented thus far indicate an increased supply of monocytes and macrophages during aging, which may be further expanded with tumor growth. Ly6C^high^ monocytes may be particularly important for the supply of TAMs to tumors. Increased availability of these cells could result in faster tumor growth, as reported in our earlier study (Duong et al., 2018). Furthermore, alterations to CSF-1R expression may impact the supply potential of these cells in terms of numbers and migration. This prompted us to investigate infiltrating monocyte/macrophages and their maturation in the tumor *via* differential expression of Ly6C and F4/80 (Ramachandran et al., 2012; Crane et al., 2014): with F4/80^int^Ly6C^high^ (Ly6C^high^) TAMs, representing early monocyte-derived TAMs. The loss of Ly6C expression is associated with maturation and therefore we identified F4/80^int^Ly6C^low^ (Ly6C^low^) TAMs as an intermediate subset, and F4/80^high^Ly6C^low/high^ (F4/80^high^) TAMs as a mature population (gating strategy shown in Figure 5A). We found that Ly6C^high^ TAMs increased significantly in the elderly early during tumor development (Figure 5B), whereas Ly6C^low^ proportions increased with aging, which was further exacerbated with tumor growth in the elderly (Figure 5C). However, F4/80^high^ TAMs expanded with tumor growth in both young and elderly and were the largest pool of TAM subsets in the tumor, particularly at later stages of tumor growth (Figure 5D). There was no difference in expression of the pro-tumoral marker CD206 in Ly6C^high^ or Ly6C^low^ TAMs during young and elderly tumor growth (Figures 5E,F respectively). In contrast F4/80^high^ TAMs from elderly tumor-bearing mice displayed increased CD206 expression with increasing tumor growth and compared to young mice (Figure 5G). Together, this may suggest a faster transition of Ly6C^high^ monocyte-derived TAMs towards Ly6C^low^ and pro-tumoral F4/80^high^ TAMs with aging. CSF-1R expression was similar between TAMs in young and elderly, however F4/80^high^ cells displayed the lowest expression of CSF-1R and intracellular CSF-1R expression was highest in Ly6C^low^ TAMs (Supplementary Figure S4A,B). This suggests CSF-1R signaling may vary across TAM subsets.

## Discussion

Monocyte and macrophage infiltration into the tumor microenvironment may be facilitated by CSF-1/CSF-1R signaling and our previous studies indicate an expansion of TAMs in elderly vs. young mice (Duong et al., 2018). Therefore, in this study we investigated the impact of aging and CSF-1R signaling on monocyte and macrophages in the bone marrow, spleen and in TAMs during tumor growth. We showed an expanded reservoir of macrophages and monocytes, specifically the Ly6C^high^ subpopulation, with aging and cancer progression. This corresponds with increased proportions of Ly6C^high^ TAMs early and Ly6C^low^ TAMs later during tumor development in the elderly. Faster maturation of these cells to F4/80^high^ TAMs may occur with aging, supported by increased CD206 expression. Furthermore, we found CSF-1R expression levels differed depending on tissue site, cell type/subset, and that this may be further impacted by age.

It is now well-established that aging leads to an increase in myelopoiesis (Rossi et al., 2005; Cho et al., 2008; Dykstra et al., 2011; Ho et al., 2017), with expansion of monocyte and macrophage numbers (Strohacker et al., 2012; Jackaman et al., 2013; Puchta et al., 2016; Duong et al., 2018). This was corroborated in the present study where we saw increased proportions of these cells in the bone marrow and spleen of healthy elderly mice. The increase in Ly6C^high^ monocytes suggests that an augmented pool of these cells can be mobilized when the need arises. Although the main site for this is thought to be the bone marrow, the spleen can provide an emergency supply of these cells if required (Cortez-Retamozo et al., 2012; Cortez-Retamozo et al., 2013).

CSF-1R expression is important for the proliferation, differentiation, maintenance, and migration of monocytes/macrophages (Reviewed in Pixley and Stanley, 2004; Yu et al., 2008; Pixley, 2012; Stanley and Chitu, 2014; Sinha et al., 2021). We did not observe any differences in expression of CSF-1R on monocytes in the bone marrow and spleen. However, in a study by Hearps et al. (2012), aging was found to be associated with decreased CSF-1R on monocytes in humans. Interestingly, we observed decreased CSF-1R in splenic macrophages. This reduction was less apparent in bone marrow macrophages, which suggests there are cell lineage- and site-specific differences. The spleen is one of the main filters for circulating blood and therefore is more likely to be impacted by the aging microenvironment compared to the bone marrow. Surface CSF-1R can be downregulated in several instances, including endocytosis due to CSF-1 ligand binding (Lou et al., 2014) and in the presence of IFN-γ (Delneste et al., 2003). Interestingly, IFN-γ can stimulate monocytes to produce CSF-1 and drive their differentiation towards macrophages (Delneste et al., 2003). Moreover, IFN-γ production was found to be upregulated with age (Bandrés et al., 2000; Singh et al., 2011). Studies have also shown that circulating levels of CSF-1 increase during aging (Suehiro et al., 1999; Larsson et al., 2015; Lira-Junior et al., 2017). In contrast, we observed no difference in CSF-1 levels during healthy aging and there was a decrease in circulating CSF-1 in elderly-tumor bearing mice. These differences between studies could potentially be due to mice strains and/or sex differences (reviewed in Duong et al., 2021) as CSF-1 is reported to be higher in males (Ramsey et al., 2012), with conflicting studies reported in females (discussed in Lira-Junior et al., 2017). Our study examined female mice and analysis of sex-specific differences in CSF-1R expression during aging requires further investigation. Overall, it is possible that aging leads to an increased potential for monocyte to macrophage differentiation, through CSF-1R signaling/increased CSF-1 utilization, that is mediated by both the aging and tumor microenvironment. This may explain the increased macrophage proportions associated with changes to CSF-1R expression during aging.

The age-related expansion of bone marrow monocyte and macrophage pools can potentially supply more infiltrating cells to a solid tumor. This may be mediated through CSF-1R signaling. Surface expression of CSF-1R was downregulated in total monocytes suggesting that aging downregulates this molecule. Interestingly, at later stages of tumor development, bone marrow Ly6C^high^ monocytes in the elderly had significantly increased intracellular CSF-1R expression compared to their younger counterparts. This was similar in bone marrow macrophages but occurred early during tumor growth. These data suggest increased CSF-1R signaling, which can contribute to the motility and egress of these cells from the bone marrow in response to tumor-derived factors.

Our study showed increased proportions of splenic monocytes in the elderly compared to young, in healthy and tumor-bearing mice. This was likewise observed in splenic macrophages; however, the impact of the tumor was greater in the elderly. The pool of macrophages in the spleen was further amplified in the elderly with tumor induction (healthy vs. early-stage tumors) and progression, whereas macrophage proportions in the young remained similar throughout. Therefore, aging drives an increased pool of monocytes and macrophages in the spleen, which is further amplified with tumor growth. Splenic Ly6C^high^ monocytes in the elderly also follow this expansion process during tumor growth, resulting in almost a tripling of proportions compared to young at late tumor growth. The increase in the availability of these cells in the elderly may be critical to cancer growth and outcomes, as splenic Ly6C^high^ monocytes are important tumor infiltrating cells (Cortez-Retamozo et al., 2013; Shand et al., 2014; de-Brito et al., 2019). However, the contribution of the spleen to the tumor during aging should be investigated further, as these studies were conducted using young mice.

The increased reservoir of monocytes and macrophages with aging and its further exacerbation with cancer in the elderly may translate to changes to TAM proportions. Indeed, we observed increased monocyte-derived Ly6C^high^ TAMs at early stages of tumor development, followed by intermediate Ly6C^low^ TAMs expanding at later stages of tumor development in the elderly compared to young. This suggests that more TAM progenitors infiltrate the tumor and mature more quickly into F4/80^high^ TAMs in the elderly. This was also supported by increased expression of the pro-tumoral marker CD206 in F4/80^high^ TAMs during aging. The highest proportion of TAM subsets in the tumor at any given stage are the F4/80^high^ cells (Figure 5), which further increase with tumor growth. The implication of this may be drawn from our previous study, showing F4/80^high^ TAMs are responsible for poorer anti-tumor immunity, as depletion of this population significantly slowed tumor growth and improved immunotherapy in the elderly (Duong et al., 2018).

CSF-1R expression may be differentially regulated in distinct TAM populations, with intracellular levels highest amongst the Ly6C^low^ subset. In contrast, F4/80^high^ TAMs had relatively lower CSF-1R expression both surface and intracellularly, which may impact their response to CSF-1R blockade therapies in cancer. In previous studies, CSF-1R inhibition was shown to target pro-tumorigenic TAMs in glioblastoma (Pyonteck et al., 2013) and pancreatic tumor models in mice (Zhu et al., 2014). Counterintuitively to our data, Zhang et al. (2020) showed in Renca tumours that F4/80^high^ expressing TAMs were preferentially depleted and F4/80^low^ TAMs increased proportionally with treatment, likely owing to differential sensitivity to CSF-1R blockade. However, another possibility is that CSF-1R inhibition targeted “earlier” TAM populations, minimizing the transition of these cells to mature F4/80^high^ TAMs and therefore reducing their proportion in the tumor. Interestingly, timing of CSF-1R inhibition can impact anti-tumor response with earlier treatment being more effective (O’Brien et al., 2021), likely due to the distribution of TAM subsets at different stages of tumor development. This may therefore have severe implications in the aging environment where differences were observed in TAM subsets and to progenitor cells in the bone marrow and spleen. Thus, future work should delineate the effects of CSF-1R inhibition on these cell populations.

In our study we described the changes to CSF-1R expression with aging and cancer in monocytes and macrophages, however the presence of CSF-1 affects surface and intracellular expression of CSF-1R. This includes receptor dimerization and autophosphorylation, initiation of downstream signaling, internalization of the receptor and its degradation *via* the lysosome pathway (Yeung and Stanley, 2003). Thus, future work could investigate CSF-1R pathway activation, receptor internalization and degradation during aging. In summary, we have shown that monocyte and macrophage populations in the bone marrow and spleen increased during healthy aging and was further impacted with cancer. CSF-1R expression in these cells were altered with age, which may impact the migration, expansion, differentiation, and survival of TAMs and, therefore, may contribute to cancer progression. There were also proportional changes to TAM subsets in elderly versus young tumors, whereby there may be a faster transition from infiltrating monocyte-derived Ly6C^high^ TAMs towards mature F4/80^high^ TAMs, with differences in CSF-1R expression. These changes may have implications towards the therapeutic effects of CSF-1/CSF-1R signaling blockade.

## Data Availability

The raw data supporting the conclusion of this article will be made available by the authors, without undue reservation.
