# Supplementary material for: Aging Leads to Increased Monocytes and Macrophages With Altered CSF-1 Receptor Expression and Earlier Tumor-Associated Macrophage Expansion in Murine Mesothelioma
Source: Front Aging. 2022 Apr 27;3:848925. doi: 10.3389/fragi.2022.848925 (PMC9261395; doi:10.3389/fragi.2022.848925)
Supplement: Supplementary file 1 [file DataSheet1.PDF]

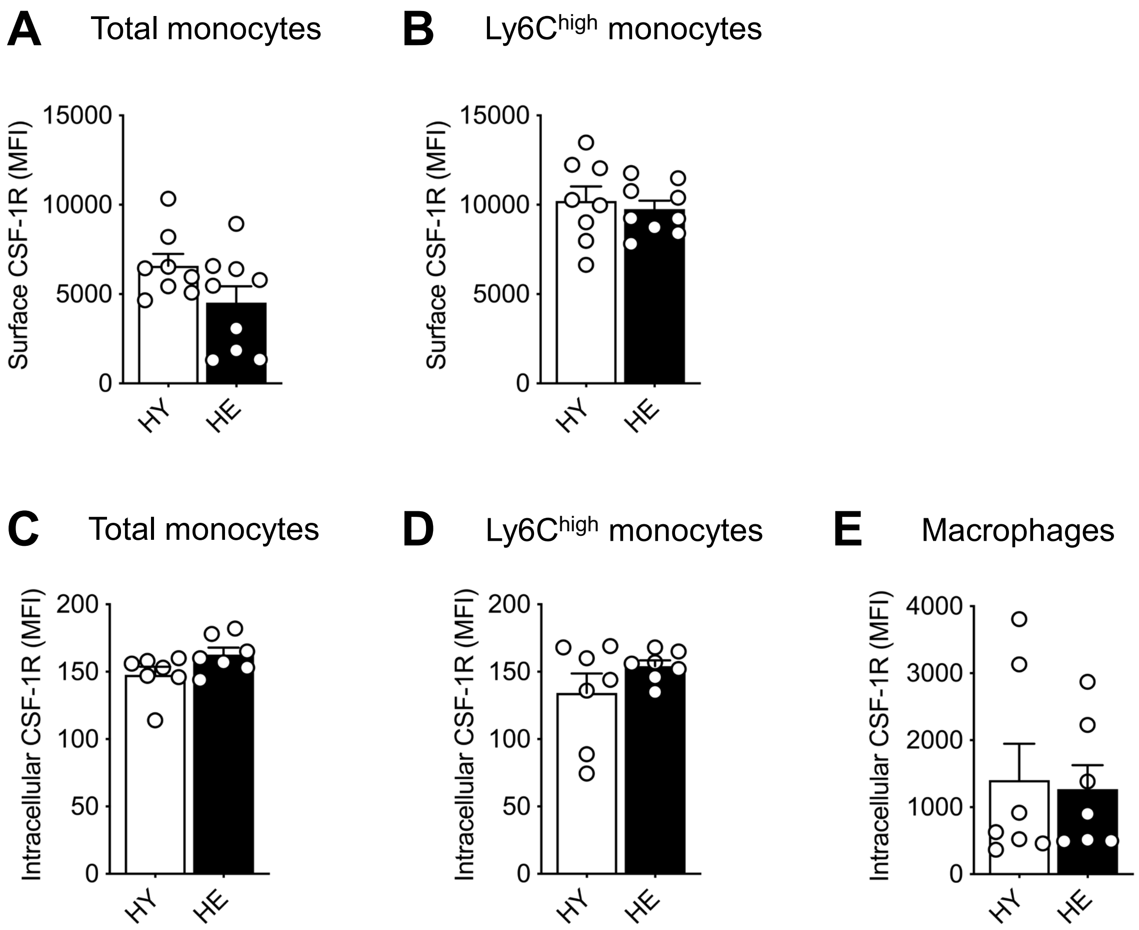

Supplementary Figure 1. Bone marrow monocyte CSF-1R expression was similar between young and elderly healthy controls. Surface CSF-1R expression was measured in total monocytes (CD11b^{+}F4/80^{neg}Ly6G^{neg}) (**A**) and in the Ly6C^{high} subpopulation of monocytes (**B**) in the bone marrow. Intracellular CSF-1R expression shown for total monocytes (**C**), Ly6C^{high} monocytes (**D**) and macrophages (CD11b^{+}F4/80^{+}Ly6G^{neg} (**E**) in the bone marrow. Expression shown as median fluorescence intensity (MFI). HY – healthy young, HE – healthy elderly. Data shown as mean ± SEM; n = 7-9 mice/group.

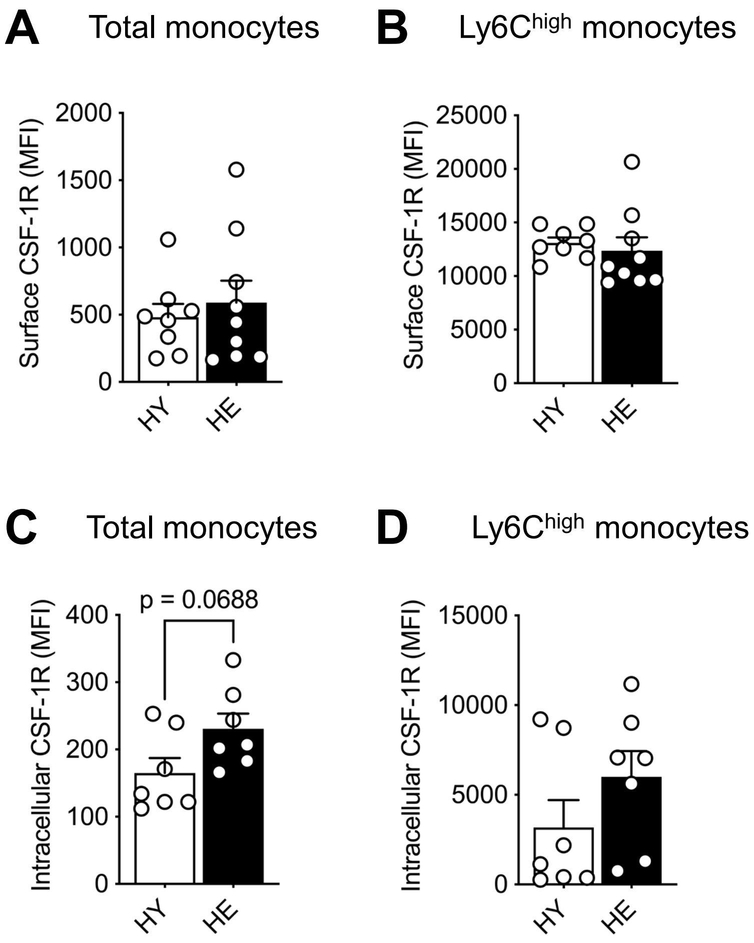

Supplementary Figure 2. Splenic monocyte CSF-1R expression was similar between young and elderly healthy controls. Surface CSF-1R expression was measured in total monocytes ($CD11b^{+}F4/80^{neg}Ly6G^{neg}$) (**A**) and in the $Ly6C^{high}$ subpopulation of monocytes (**B**) in the spleen. Intracellular CSF-1R expression shown for total monocytes (**C**), and $Ly6C^{high}$ monocytes (**D**) in the spleen. Expression shown as median fluorescence intensity (MFI). HY – healthy young, HE – healthy elderly. Data shown as mean ± SEM; n = 7-9 mice/group.

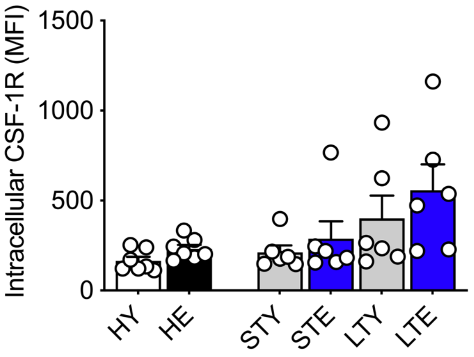

Supplementary Figure 3. Splenic monocyte intracellular CSF-1R expression was similar between young and elderly AE17 tumor-bearing mice. Intracellular CSF-1R expression was measured in total monocytes ($CD11b^{+}F4/80^{neg}Ly6G^{neg}$). Expression shown as median fluorescence intensity (MFI). HY – healthy young, HE – healthy elderly, STY – small tumor young, STE – small tumor elderly, LTY – large tumor young, LTE – large tumor elderly. Data shown as mean ± SEM; n = 6-7 mice/group.

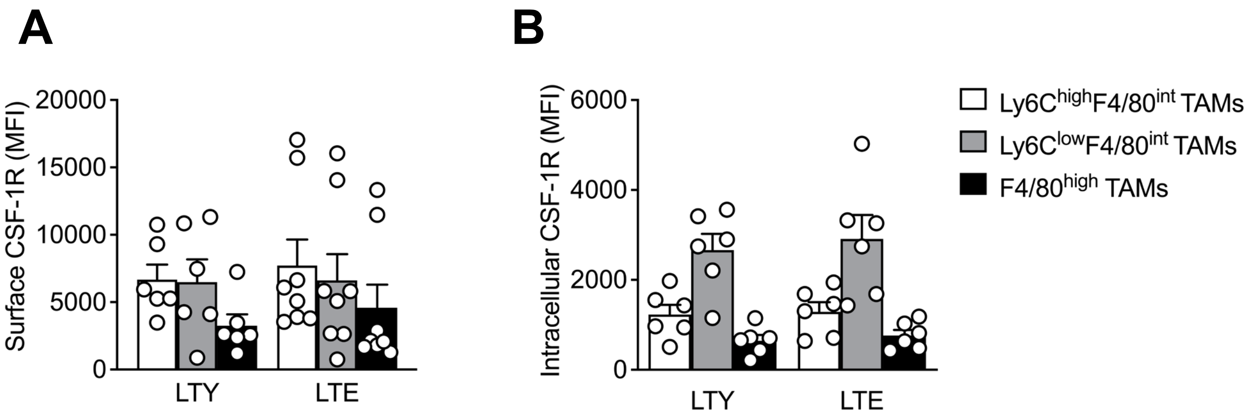

Supplementary Figure 4. Ly6C^{low} tumor-associated macrophage (TAMs) exhibited the highest expression intracellular CSF-1R. CSF-1R shown as surface (A) and intracellular (B) levels expression was measured in TAM (CD11b^{+}F4/80^{+}Ly6G^{neg}) subsets. Expression shown as median fluorescence intensity (MFI). LTY – large tumor young, LTE – large tumor elderly. Data shown as mean ± SEM; n = 6-9 mice/group.
